# Knowledge, Awareness, and Practice Towards the Use of *Salvadora persica* L. (Miswak) Chewing Stick: A Scoping Review

**DOI:** 10.3390/healthcare13212747

**Published:** 2025-10-30

**Authors:** Nurul Fatin Azizan, Nurulhuda Mohd, Nik Madihah Nik Azis, Badiah Baharin

**Affiliations:** 1 Oral Health Programme, Ministry of Health, Putrajaya 62590, Malaysia; 2 Department of Restorative Dentistry, Faculty of Dentistry, Universiti Kebangsaan Malaysia, Jalan Raja Muda Abdul Aziz, Kuala Lumpur 50300, Malaysia

**Keywords:** behavior, dental public health, oral hygiene, perception, Siwak

## Abstract

**Background:** The benefits of *Salvadora persica* L. chewing stick as an oral hygiene tool have been extensively demonstrated in clinical studies worldwide. Nonetheless, there are wide variations in knowledge, awareness, and practice of these chewing sticks across different populations. This scoping review aims to synthesize current knowledge gaps and practice patterns to inform potential standardization of *S. persica* use. **Methods:** Following PRISMA-ScR guidelines, a systematic literature search was conducted by using the Web of Science, Medline, and Scopus databases, covering studies published up to June 2025. **Results:** Twenty-seven studies were included, involving diverse populations from Africa, the Middle East, and Asia. Knowledge, awareness, and practice of *S. persica* chewing sticks varied significantly by region, demographic group, and mode of use. Most studies evaluating awareness and knowledge reported that a lack of information on proper use leads to reduced practice. There was no standardized method of use reported. Behaviors varied in terms of preparation of the stick before use, frequency and duration of use, angle or technique during brushing, and storage methods after use. **Conclusions:** The findings emphasize the need for culturally sensitive clinical guidelines and community health education programs to inform both the public and healthcare professionals about the use of *S. persica* chewing sticks–particularly in populations with limited access to other oral hygiene tools.

## 1. Introduction

Maintaining good oral hygiene is essential for both optimal oral health and overall well-being. Knowledge and awareness of oral health are key factors in encouraging positive health-related behavior [1]. While evidence from cross-sectional studies shows only a weak association between knowledge and behavior [2], individuals with better awareness and understanding of oral health are generally more likely to engage in proper self-care practices [3]. A positive attitude toward oral hygiene also plays a crucial role, as it motivates and drives effective personal oral care routines [4]. The prevalence of oral diseases, namely dental caries and periodontal diseases, tends to decrease with improved oral hygiene practice, highlighting the importance of individual behavior [5]. Thus, a combination of adequate knowledge, awareness, and consistent oral hygiene practice are instrumental in the pursuit of good oral health.

Regular removal of bacterial plaque is the most effective method for preventing caries and periodontal disease [6]. This is commonly accomplished through standard toothbrushing, often supplemented with chemical adjuncts such as toothpaste or mouth rinses [7]. However, oral care practices vary across the world, influenced by diverse cultural customs and traditional beliefs. Despite enormous advances in modern dental science, plants have continued to serve as a significant natural source for oral hygiene in many regions [8]. One such example is the *Salvadora persica* chewing stick, commonly known as miswak, which has been used for plaque removal for centuries [8]. The Arabic term “miswak” refers to a chewing stick made from the roots, stems, twigs, or bark of the *S. persica* L. plant [9], also known as the “toothbrush tree” [10]. This traditional practice continues in many communities worldwide despite the widespread modern use of the standard toothbrush [11,12]. In addition to being widely available and low cost in many countries, the use of *S. persica* chewing sticks remains especially common among Muslims as part of their domestic oral hygiene habits [13]. As such, religion is believed to play a significant role in the widespread use of *S. persica* chewing sticks as a cultural and religious oral hygiene practice across geographical boundaries [14].

Unfortunately, contemporary dietary habits have shifted dramatically due to the widespread availability and consumption of industrially processed foods. These foods are often high in added sugars and artificial ingredients designed to enhance flavors and prolong shelf life [15]. These additives, particularly sugars, have been strongly linked to the increased prevalence of dental caries and periodontal disease [15]. The contrast between the traditional use of *S. persica* chewing sticks and the modern reliance on processed foods highlights a broader transformation in both lifestyle and health priorities. Beyond their cultural and religious significance, *S. persica* chewing sticks are valued for their natural antibacterial properties, which help clean the teeth, freshen the breath, and even support periodontal health [14,16,17]. Unlike modern toothbrushes, which require toothpaste, *S. persica* chewing sticks offer a natural, cost-effective alternative that aligns with holistic health practices [16,17].

Although studies on the effectiveness of *S. persica* chewing sticks have reported mixed results globally, a recent systematic review and meta-analysis concluded that miswak chewing sticks are as effective as standard toothbrushes in reducing plaque, and even more effective in preventing gingivitis [16]. Researchers attribute the therapeutic benefits of *S. persica* chewing sticks primarily to their mechanical cleaning action [17], further enhanced by the release of biologically active compounds or the combined mechanical and chemical effects [17]. This high level of evidence highlights the effectiveness of *S. persica* chewing stick as a viable alternative to toothbrush for promoting oral health [18,19]. Moreover, the World Health Organization advocates the use of *S. persica* chewing sticks as an effective oral hygiene tool, particularly in areas where their use is customary [20]. This approach is also regarded as equitable, efficient, and cost-effective, aligning with the principles of primary health care [20,21].

The Consensus Statement on Oral Hygiene [22] emphasizes that chewing sticks are among the most effective tools for plaque control, provided they are used thoroughly and on a daily basis [18]. Nevertheless, there remains a lack of information about public knowledge and awareness regarding the use of chewing sticks as an oral hygiene tool, which may hinder their effective practice. Additionally, no standardized guidelines currently exist to define correct usage techniques or address the limitations associated with chewing stick use [16]. This is reflected in the diverse methods of use reported across different studies and populations. In some cases, individuals use chewing sticks in combination with a standard toothbrush rather than as a standalone tool [23]. Thus, this review aims to provide an overview of existing studies on the knowledge, awareness, and oral hygiene practices related to the use of *S. persica* chewing sticks.

## 2. Materials and Methods

### 2.1. Ethical Approval

The research presented did not involve human or animal subjects; therefore, ethical approval was not applicable to this review.

### 2.2. Review Registration

The protocol for this scoping review has been registered with the Open Science Framework (OSF; registration number: osf-registrations-gaz9w-v1).

### 2.3. Search Strategy

This review follows the established methodological framework outlined by the Preferred Reporting Items for Scoping Reviews (PRISMA-ScR) to ensure rigor and clarity [24]. The research questions guiding this review are: (i) What is the level of knowledge and awareness regarding the use of *S. persica* chewing sticks among populations? (ii) How do knowledge and awareness of *S. persica* chewing stick influence its practice behavior?

A literature search was conducted for publications up to June 2025 across three databases: Medline, Scopus, and Web of Science. The search terms used were: (“*Salvadora persica*” [Mesh] OR “*Salvadora persica*” OR “Miswak” OR “Miswaak” OR “Siwak” OR “Sewak” OR “Chewing stick” OR “Natural toothbrush”) AND (“Knowledge” OR “Awareness” OR “Perception” OR “Attitude” OR “Practice” OR “Behavior” OR “Use” OR “Utilization” OR “Oral hygiene practice” OR “Dental hygiene practice”). Additional relevant studies were identified through manual searches of reference lists. Unpublished data were excluded to maintain consistency throughout the screening and selection process. The search was restricted to articles published in the English language due to resource constraints, with no limitations on the publication year. This restriction is acknowledged as a limitation, particularly regarding culturally diverse practices, as relevant studies published in other languages may have been excluded.

### 2.4. Study Selection

The initial screening of identified data was conducted based on titles and abstracts by two independent reviewers (N.F.A. and N.M.N.A.). Full texts of potentially eligible studies were then retrieved and assessed for suitability according to the predefined inclusion and exclusion criteria. Any disagreements between the reviewers regarding study selection were resolved through discussion with a third reviewer (N.M.).

The inclusion criteria were defined based on the Participant/Population (P): *S. persica* chewing stick users; Concept (C): knowledge, awareness, and practice behavior of the use of *S. persica* chewing sticks; Context (C): the use of *S. persica* chewing stick in its raw form. Studies were excluded if they were case reports, case studies, in vitro studies, reviews, editorials, letters to editors, or expert opinions. Articles that solely reported the prevalence of *S. persica* chewing stick use were also excluded.

### 2.5. Quality Assessment, Data Extraction and Analysis

As most of the included articles employed self-reported questionnaires, a methodological quality appraisal was performed as an optional step to provide additional context for interpreting the evidence [25]. The Joanna Briggs Institute (JBI) Critical Appraisal Checklist for Analytical Cross-Sectional Studies was used for this purpose [26,27]. The quality assessment was evaluated by N.F.A. and N.M.N.A., which any disagreements were resolved through discussion until consensus was reached. All cross-sectional studies were included in this review regardless of their quality or risk-of-bias outcomes. No critical appraisal was conducted for qualitative studies, as the focus was on identifying recurring themes related to knowledge, awareness, and practices rather than evaluating methodological rigor.

Data extraction and synthesis were carried out by the first reviewer (N.F.A.) and verified by the second reviewer (N.M.N.A.) to ensure consistency with the research questions. The extracted information from the included studies was summarized and presented in a table of evidence. The data extraction parameters included: primary author, year of publication, study location/country, study design, population setting, and findings related to knowledge, awareness, and practice behaviors concerning *S. persica* chewing sticks.

## 3. Results

### 3.1. Identification of Potential Studies

**Figure 1:**
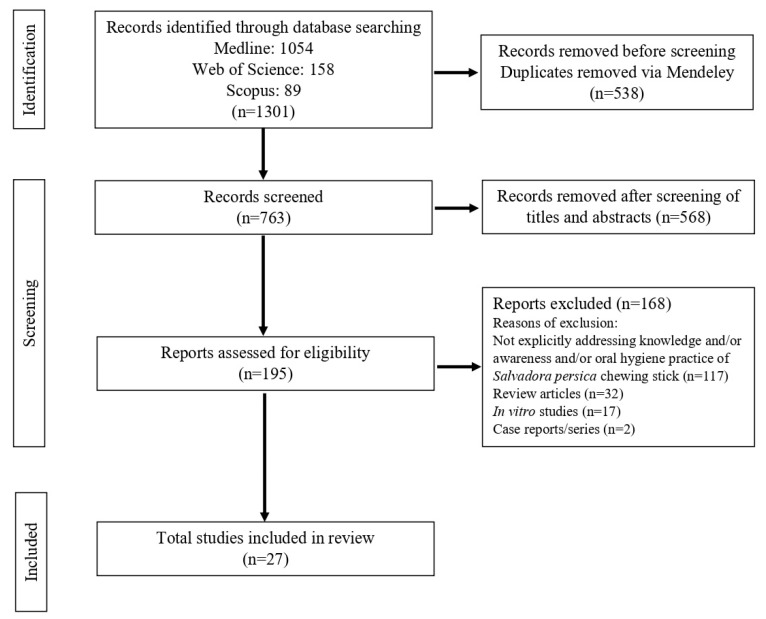
PRISMA flow diagram depicting the results of the search strategy.

The literature search identified 1301 relevant articles related to public knowledge, awareness, and practice. Based on the predefined criteria, two case reports/series, seventeen in vitro studies, and thirty-two review articles were excluded. Additionally, 117 studies were removed as they did not specifically address the knowledge, awareness, and practice of *S. persica* chewing sticks, focusing instead on their clinical benefits. After screening titles and abstracts, followed by full-text assessment, 27 studies were ultimately included in this review (Figure 1).

### 3.2. Characteristics of the Included Studies

The 27 included articles were published between 1995 and 2023. Most studies were conducted in the Middle East (n = 13) [3,13,23,28,29,30,31,32,33,34,35,36,37], while others originated from Africa (n = 6) [38,39,40,41,42,43], South Asia (n = 4) [44,45,46,47], Southeast Asia (n = 3) [48,49,50], and Oceania (n = 1) [51].

Among these studies, 10 were qualitative, comprising one focus group discussion [48] and nine structured interviews [30,34,35,39,41,42,46,47,51]. The remaining 17 were cross-sectional studies [3,13,23,28,29,31,32,33,36,37,38,40,43,44,45,49,50]. The critical appraisal of the included cross-sectional studies is presented in Table 1.

Most studies focused on students (n = 11) [3,23,32,34,35,36,37,41,43,44,50], with participant groups ranging from primary school children to college/university students. Three studies investigated the general adult population [13,33,49], while two studies included both adults and children/adolescents [42,46]. Six studies focused on patients attending dental clinics [28,30,32,37,45,47]. Five studies examined specific groups, including schoolteachers [29], dental educators [48], visually impaired individuals [39], Muslim inhabitants [38], and immigrants [45].

Seven studies explored the relationship between *S. persica* chewing stick use and demographic variables in terms of age and gender [23,30,33,34,37,38,42]. Across six studies, the majority of *S. persica* chewing stick users were reported to be male [23,30,33,34,37,38], whereas only one study found a predominance of female users [42]. Age-related patterns varied: one study reported no significant difference in usage [37], while two indicated an increase in use with age [30,38]. Socioeconomic disparities were also observed, encompassing variations in education level, income, and the geographical location of schools (rural versus urban areas) [30,31,42,43,45,47]. While one study found no significant association between education or income and the selection of oral hygiene tools [29], two studies highlighted a notably higher proportion of chewing stick users in rural areas compared to urban settings [42,43]. Three studies linked chewing stick use primarily to individuals with primary education and those employed in unskilled occupations [30,43,47]. Interestingly, one study reported that users who combined chewing sticks with toothbrushes were more likely to hold bachelor’s or associate degrees [31].

Most data were collected using self-administered questionnaires (n = 17) [3,13,23,28,29,32,33,36,37,38,40,42,43,44,45,49,50]. Nine studies employed structured interviews with pre-designed questionnaires [30,34,35,39,40,41,46,47,51], while one study utilized a focus group discussion [48]. The detailed characteristics of the included studies are summarized in Table 2 and Supplementary Table S1.

### 3.3. Definition of Knowledge, Awareness, and Oral Hygiene Practice

In this review, knowledge, awareness, and oral hygiene practice were assessed in relation to the use of *S. persica* chewing sticks.

Knowledge was defined as understanding the benefits and proper use of chewing sticks as an oral hygiene tool for oral disease prevention and/or oral health care [28,48,49]. Awareness reflected participants’ reasons and perceptions regarding the use of chewing sticks, particularly in relation to cultural or religious familiarity [3,13,23,29,30,31,32,38,49,50]. However, knowledge and awareness were used interchangeably in several studies [13,30,31,38,50,51], particularly in the context of religious purpose for chewing stick use [13,30,38,51]. Consequently, the results in this review were reported according to how the original studies defined these terms.

Oral hygiene practice was reported based on how the participants used *S. persica* chewing stick—whether as their primary oral hygiene tool or in combination with other tools [3,13,23,30,31,32,38,44]. Practice behavior was further characterized by oral hygiene habits such as the methods of use, duration, age at the start of use, frequency and/or time of use, and storage practices after use. Variations in practice behavior related to age, gender, and socioeconomic background were also reported when available in the included studies [23,28,29,30,32,33,34,36,37,45,47].

### 3.4. Knowledge

Among all the studies analyzed, only three specifically focused on evaluating knowledge regarding the use of *S. persica* chewing sticks [28,48,49]. In the study by Almas et al., 2000, the assessment addressed knowledge of oral diseases prevention methods [28]. Over two-thirds of the participants reported had knowledge of the use of *S. persica* chewing sticks for this purpose [28]. Che Musa et al., 2020 reported that the limited understanding of the efficacy of *S. persica* chewing sticks for oral hygiene was attributed to the scarcity of comprehensive and widely available scientific evidence [48]. Furthermore, survey respondents concurred that the lesser practice of *S. persica* chewing stick stemmed from inadequate information about its proper use [48]. Nordin et al., 2014 uncovered that most participants had knowledge of the oral health benefits of using *S. persica* chewing sticks [49]. Over 90% of respondents recognized that religious factors influenced their practice. However, the study also highlighted a lack of detailed understanding among respondents regarding the proper methods for using *S. persica* chewing sticks [49].

### 3.5. Awareness

Fourteen studies examined awareness of chewing stick use [3,13,23,29,30,31,32,38,39,44,45,49,50,51]. Awareness was reported in terms of perceived reasons and perception of *S. persica* chewing stick use, as well as views on recommending their use to future generations [13,32].

Ten studies documented the perceived reasons for using chewing sticks [3,13,29,30,32,38,39,44,45,51]. These included religious motivations [13,29,30,32,38,39,44,45,51], cultural practices [32,44], traditional beliefs [3], availability [32], practicality [39,44], and scientific support [13,32,38,44,51]. Oral hygiene-related reasons were also cited [30], such as maintaining fresh breath [13,29,32], achieving whiter teeth [13,32], and improving cleaning efficacy compared with other oral hygiene tools [3,29,31,32,38,39,45]. Several studies explored respondents’ awareness of the benefits of chewing sticks for maintaining and improving oral health [33,34,45,49,50,51]. Most respondents were aware of the effectiveness of *S. persica* chewing sticks in reducing plaque levels [30,50,51]. In addition, two studies reported that respondents recommended the combined use of chewing sticks and toothbrushes as part of daily oral hygiene routine for future generations [13,32].

Interestingly, the level of education added a distinct perceptive to the awareness of chewing stick use. ALGhamdi et al., 2015 found that, despite receiving oral hygiene education, dental students did not consider the *S. persica* chewing stick to be the sole armamentarium for teeth cleaning [23]. In contrast, Tubaishat et al., 2005 observed that dental patients with higher education perceived the combination of a toothbrush and chewing stick as the most effective method for plaque reduction [31].

### 3.6. Oral Hygiene Practice

A total of twenty-five studies (n = 25) assessed oral hygiene practices related to chewing stick use among participants [3,13,23,28,29,30,31,32,33,34,35,36,37,38,39,40,41,42,43,44,45,46,47,50,51]. The reported practices varied considerably across studies. Fourteen studies (n = 14) specifically examined whether *S. persica* chewing sticks were used as the primary oral hygiene tool or in combination with other oral care aids [13,23,29,30,31,32,39,40,41,44,45,46,50,51]. The prevalence of using *S. persica* chewing sticks as the sole cleaning tool ranged from 2.3% to 44.0% [13,29,30,31,32,41,44,45,50,51]. However, most participants reported combining *S. persica* chewing sticks with standard toothbrushes [13,29,30,31,32,40,44,46,50,51].

The documented oral hygiene practices covered various aspects, including the frequency or timing of use [3,28,29,31,33,36,39,45], duration of use [3,31,32,39], method or angle of use [28,29,51], and storage practices after use [13,32,44]. One study noted that respondents used *S. persica* chewing sticks not only for tooth cleaning but also for tongue cleaning [40]. Another study reported no significant gender differences in the methods of chewing stick use (*p* = 0.189), nor were there significant differences in the frequency of daily use across different income groups (*p* = 0.382) [29].

Overall, no standardized practice pattern for chewing stick use was observed across the included studies. A summary of the findings is presented in Table 3.

## 4. Discussion

To the best of our knowledge, this is the first scoping review to provide an overview of knowledge, awareness, and practices related to the use of *S. persica* chewing sticks. Generally, more than 70% of respondents demonstrated knowledge of *S. persica* chewing sticks as a preventive tool against oral diseases. Although respondents recognized their potential benefits in maintaining oral health, many lacked proficiency in their proper use as an effective oral hygiene tool. This knowledge gap appears to stem from the limited and localized nature of existing evidence [28,48,49], further compounded by the scarcity of studies specifically investigating respondents’ understanding of correct *S. persica* chewing stick usage for optimal oral health maintenance.

Oral health literacy among *S. persica* chewing sticks users appears to be primarily shaped by personal beliefs, cultural traditions, and educational exposure. Interestingly, this traditional practice continues even among postmodern communities in the Middle East, Africa, and South Asia [31]. Central to its continued use is the perceived efficacy of the chewing sticks, which strongly influences users’ attitudes and intentions regarding its use [51]. The high prevalence of *S. persica* chewing stick use in rural communities [42,43] may be attributed its easy accessibility and the ability to produce it from locally available twigs, roots, or branches without incurring financial cost [38]. Although the use of *S. persica* chewing sticks predates the advent of Islam, it has since become a highly endorsed oral hygiene practice within Islamic teachings [14]. In many Muslim-majority regions, religious belief serves as a strong motivational factor, influencing the use of *S. persica* chewing sticks not only as a means of oral hygiene but also as a reflection of religious devotion and cultural identity [11,52]. Moreover, many chewing stick users express a preference for their children to incorporate chewing sticks as part of their oral hygiene routine [13,32]. Within religious households and schools, this tradition is often embedded in familial and communal norms, where children learn the values of chewing sticks through the lens of spiritual teachings on cleanliness and discipline [52].

Nonetheless, as global populations become increasingly diverse in their social, religious, and cultural backgrounds, it is essential for healthcare professionals to develop cross-cultural competence to better understand accepted societal norms and health-related behaviors [18]. In regions where *S. persica* chewing stick use is customary, it should be recognized as a culturally appropriate oral hygiene practice rather than merely a substitute for a regular toothbrush [53]. Dental practitioners should also acknowledge the scientific validity supporting *S. persica* use, as doing so can foster positive dentist-patient relationships and ultimately enhance public oral health outcomes [18,51].

Therefore, particular attention should be directed toward the knowledge and awareness of dental students, who are future professionals in the field, as well as their educators [18]. This review highlights a notable deficiency in scientific knowledge regarding the effectiveness of *S. persica* chewing stick, which has led to misconceptions about its efficacy [48] and reinforced the belief that *S. persica* chewing sticks should not be solely relied upon for maintaining oral hygiene [52]. Furthermore, the lack of structured health education on the proper use and benefits of *S. persica* chewing sticks within dental training may compromise the quality of oral health guidance provided to patients [48]. A key contributing factor to this knowledge gap is the limited integration of *S. persica* chewing sticks into dental curricula, where conventional oral hygiene methods continue to dominate both education and clinical practice [48,51]. Consequently, even in regions where *S. persica* chewing stick use is customary, dental students often receive minimal exposure to the historical, cultural, and scientific foundations supporting its use. This curricular omission may inadvertently diminish the perceived value of this traditional practice as a viable oral hygiene tool [48]. Given the dynamic interplay between knowledge and awareness, a concurrent lack of both significantly impacts individuals’ attitudes that contributes to their reluctance in adopting *S. persica* chewing sticks as part of dental care practices [18].

Besides that, the non-accommodative design of raw *S. persica* chewing sticks poses challenges in terms of maneuverability during toothbrushing. This limited adaptability may be perceived as a social disadvantage in modern societies, potentially hindering their widespread use among younger generations [52]. Nevertheless, research indicates that *S. persica* chewing sticks are as effective as standard toothbrushes for plaque removal when employed correctly [16,17]. Beyond the mechanical action of these chewing sticks, the natural bioactive components released during use may confer additional chemical benefits in reducing gingival inflammation [16,54]. Comparative studies have demonstrated that habitual miswak users tend to exhibit a lower need for periodontal treatment and better periodontal status than individuals using standard toothbrushes [14]. However, excessive scrubbing of the chewing sticks can lead to adverse effects, such as occlusal tooth wear and gingival recession [52,55]. Similarly, using dry chewing sticks may damage hard tissues and traumatize the gingiva [52]. The structural design of *S. persica* chewing sticks, with bristles aligned longitudinally to the handle, also presents physical challenge during brushing [1]. Therefore, enhancing knowledge and awareness among both users and dental healthcare providers regarding proper techniques for *S. persica* chewing stick use is critical to promoting safe and effective oral hygiene practices [17,52]. This review thus emphasizes the important role of dental professionals in delivering evidence-based information about the benefits and limitations associated with the use of *S. persica* chewing sticks, to ensure their correct application and optimize oral health outcomes [54].

Given that the *S. persica* chewing stick is a technique-sensitive oral hygiene tool, there is a clear need for standardized reporting and comprehensive guidelines on its proper use to minimize potential risks. This protocol should encompass clear instructions on: (i) preparation prior to use, (ii) correct brushing technique, frequency, and duration, (iii) appropriate post-use care and storage, and (iv) necessary precautions to prevent improper use. To enhance public understanding and promote correct application, practical tools such as instructional pamphlets featuring clear diagrams and step-by-step guidance should be implemented [16]. These initiatives could guide the formulation of targeted public health policies and culturally sensitive clinical guidelines, particularly for communities with limited access to standard toothbrushes.

This scoping review has several limitations. Firstly, it only included articles published in English. The exclusion of non-English literature, particularly studies published in Arabic or other regional languages, may have led to the omission of valuable culturally relevant data and insights. Given that the use of *S. persica* chewing sticks is deeply rooted within specific cultural and religious traditions, relevant research published in local or regional journals may not have been included. This language restriction introduces a potential source of selection bias, which could affect the comprehensiveness and cultural representativeness of the findings. As a result, this limitation should be carefully considered when interpreting the results and assessing the applicability of the conclusions to broader or more diverse populations.

Secondly, although this review includes both survey-based and qualitative studies, it did not employ a formal qualitative appraisal tool, nor did it present a dedicated qualitative synthesis, as these were beyond the scope of the review. Future reviews could adopt a more focused qualitative approach, particularly as the body of relevant qualitative research continues to grow. In addition, most of the included studies were cross-sectional in design and relied on self-reported questionnaires, which inherently limit external validity. These studies also provided only a cross-sectional perspective on participants’ knowledge, perceptions, or behaviors related to this oral hygiene practice, without tracking changes or outcomes over time. In addition, none of the studies conducted a comparative analysis evaluating the effectiveness of *S. persica* chewing sticks in relation to other oral hygiene tools in preventing caries and gingivitis. Nevertheless, this omission was intentional, as including clinical outcome data would have shifted the research focus from exploring perceptions and behavioral patterns to assessing clinical effectiveness, thereby mixing two distinct areas of investigation.

Additionally, many studies investigating *S. persica* chewing stick use have predominantly focused on children, adolescents, or religious participants. These groups are often selected for their accessibility, which facilitates data collection logistics. While these groups offer valuable insights, their predominance in the literature may not accurately represent the general population. Moreover, usage patterns among children and adolescents may differ substantially from those of adults or older individuals in terms of technique, frequency, and underlying motivations, such as cultural versus religious adherence [56]. Similarly, research centered on religious participants, typically Muslims who use miswak in accordance with Islamic teachings, may introduce bias. These individuals might employ miswak primarily as part of their religious observance rather than solely for oral hygiene purposes, potentially influence both the manner and frequency of use [57].

Another important limitation of this review is the localized nature of *S. persica* chewing stick use. The practice is not globally widespread but is predominantly confined to certain cultural, religious, and geographic contexts where it holds traditional significance. This contextual specificity limits the generalizability of the findings to broader and more diverse populations that do not share similar oral hygiene traditions. Consequently, the practicality and relevance of *S. persica* chewing sticks as an oral hygiene tool may not be limited in regions where the practice is not culturally embedded. Additionally, this lack of external validity may skew interpretations of the usage patterns among secular or non-religious users, who may adopt *S. persica* chewing sticks only sporadically or alongside modern oral hygiene tools. Therefore, this context-dependent nature should be acknowledged when interpreting the findings and considering their potential integration into global oral health strategies. Future research should aim to include more heterogeneous samples encompassing diverse age groups, cultural backgrounds, and both urban and rural populations to enhance generalizability and applicability.

## 5. Conclusions and Public Health Implications

There were notable variations in the knowledge, awareness, and practices related to the *S. persica* chewing sticks across different populations. Overall, the use of *S. persica* chewing stick was more prevalent among individuals with strong religious and cultural affiliations, whereas limited knowledge and awareness emerged as key barriers to its use. Although *S. persica* chewing sticks are often recommended as an alternative or complementary tool to standard toothbrushes, evidence suggests that their optimal benefits are achieved only when used regularly with proper techniques. Given the cultural specificity of *S. persica* use, the findings of this review should be interpreted preliminary and context dependent. Any recommendations or public health strategies derived from these findings should therefore be tailored to regions or populations where chewing stick use is culturally accepted and commonly practiced. The results may support the development of community-based oral health initiatives that incorporate culturally informed education and support the evidence-based integration of traditional practices into dental training. Such efforts could advance a more holistic and culturally sensitive approach to oral health care. However, broader policy adoption in regions where this practice is unfamiliar should be approached with caution and pursued only after further research validates its relevance, safety, and acceptability within those populations.

## Figures and Tables

**Table 1:** Quality assessment tool for cross-sectional studies: Joanna Briggs Institute (JBI) Critical Appraisal Checklist for Analytical Cross-Sectional Studies [27].

Authors	Clear Inclusion Criteria	Study Subjects and Setting Described	Valid and Reliable Exposure Measured	Objective, Standard Criteria Used for Measurement	Confounding Factors Identified	Assessment of Outcome	Valid and Reliable Outcomes Measured	Appropriate Statistical Analysis	Quality Appraisal
Tubaishat et al., 2005 [31]	*	*	–	*	–	*	*	*	6/8
Vanka et al., 2021 [32]	*	*	–	*	–	*	*	*	6/8
Al-Shammari et al., 2007 [33]	*	*	–	*	*	*	–	*	6/8
Darout et al., 2005 [36]	–	*	*	*	–	*	*	*	6/8
Farsi et al., 2004 [37]	*	*	–	*	–	*	*	*	6/8
Hyder et al. 2023 [39]	*	*	–	*	*	*	–	*	6/8
Varenne et al., 2006 [42]	–	*	*	*	–	*	*	*	6/8
Nordin et al., 2014 [49]	*	*	–	*	–	*	*	*	6/8
Darout et al., 2016 [3]	–	*	–	*	–	*	*	*	5/8
ALGhamdi et al., 2015 [23]	–	*	–	*	–	*	*	*	5/8
Gul et al., 2022 [51]	*	*	–	–	–	*	*	*	5/8
Almas et al., 2003 [29]	–	*	–	*	–	*	*	*	5/8
Agbor and Azodo, 2013 [38]	–	*	–	*	–	*	–	*	4/8
Azodo et al., 2010 [40]	*	*	–	–	–	*	–	*	4/8
Bramantoro et al., 2018 [50]	*	*	_	–	_	*	–	*	4/8
Al-Hammadi et al., 2018 [13]	–	–	–	–	–	*	*	*	3/8
Almas et al., 2000 [28]	–	*	–	–	–	*	–	*	3/8

*: Yes; –: No/Unclear/Not Applicable [Refer to Additional File].

**Table 2:** Characteristics of the studies included.

Authors and Year of Publication	Study Design	Study Region, Country	Method of Data Collection Related to Miswak	Number of Participants and Study Settings	Age of Participants	Percentage of Miswak Users in Study	Reported Outcome(s) Measured Related to Miswak
Che Musa et al., 2020 [48]	Qualitative	Kuantan, Malaysia	Focus-groups discussion	11 dental educators; university	NA	NA	Knowledge
Nordin et al., 2014 [49]	Cross-sectional	Kuala Lumpur, Malaysia	Self-administered questionnaires (close-ended)	517 adults; Kuala Lumpur residence	20–39 years old	NA	Knowledge; Awareness
Almas et al., 2000 [28]	Cross-sectional	Makkah, Riyadh, Tabuk and Gizan, Saudi Arabia	Self-administered questionnaires	367 dental patients; six cities in Saudi Arabia	30 ± 11.9 years old	NA	Knowledge; OH practice
Darout et al., 2016 [3]	Cross-sectional	Jazan, Saudi Arabia	Self-administered questionnaires	499 students; five secondary school (three urban and two suburban)	Grouped into 15–17 and >18 years old	All miswak users	Awareness; OH practice
Al-Hammadi et al., 2018 [13]	Cross-sectional	Aseer, Saudi Arabia	Self-administered questionnaires (online)	2023 adults; study setting NA	20–65 years old	8.0% used miswak only, 44.5% used both miswak and toothbrush	Awareness; OH practice
ALGhamdi et al., 2015 [23]	Cross-sectional	King Abdulaziz University, Saudi Arabia	Self-administered questionnaires	300 dental students; university	20.1 ± 1.6 years old	22.7% used miswak	Awareness; OH practice
Almas et al., 2003 [29]	Cross-sectional	Riyadh, Saudi Arabia	Self-administered questionnaires	470 teachers; primary and secondary schools	NA	5.6% used miswak only, 50.9% used both miswak and toothbrush	Awareness; OH practice
Al-Otaibi and Angmar-Mansson, 2004 [30]	Qualitative	Makkah, Saudi Arabia	Structured interview	1155 dental outpatients; two dental centers	10–60 years old	17.0% used miswak only, 73.2% used both miswak and toothbrush	Awareness; OH practice
Tubaishat et al., 2005 [31]	Cross-sectional	Irbid, Jordan	Self-administered questionnaires	138 adults; one public and two private dental clinics	18–60 years old	3.0% used miswak only, 20.5% used both miswak and toothbrush	Awareness; OH practice
Vanka et al., 2021 [32]	Cross-sectional	Saudi Arabia	Self-administered questionnaires (open-ended)	342 adults; private dental hospital	Mean age 31	2.3% used miswak only, 31.6% used both miswak and toothbrush, 40.5% used miswak whenever required	Awareness; OH practice
Agbor and Azodo, 2013 [38]	Cross-sectional	Banyo in Adamawa region of Cameroon	Self-administered questionnaires	220 adult Muslims; Banyo residence	Mean age 28 (21–50 years old)	85.0% used miswak	Awareness; OH practice
Hyder et al., 2023 [39]	Cross-sectional	Karachi, Pakistan	Self-administered questionnaires	530 adults; dental outpatient department of a university	18–68 years old	5.8% used miswak only, 23.2% used both miswak and toothbrush	Awareness; OH practice
Fantaye et al., 2022 [44]	Qualitative	Addis Ababa, Ethiopia	Structured interview	45 totally visually impaired and 20 partially visually impaired individuals; study setting NA	Mean age 27.2 (10–65 years old)	57.7% totally and 30.0% partially visually impaired used miswak, 35.5% and 55.0% partially visually impaired used both miswak and toothbrush	Awareness; OH practice
Alayan et al., 2017 [45]	Qualitative	New Zealand	In-depth semi-structured interviews	8 Muslim immigrants; study setting NA	NA	All miswak users	Awareness; OH practice
Bramantoro et al., 2018 [50]	Cross-sectional	Surabaya, Indonesia	Self-administered questionnaires Characteristic assessment: semi-open Behavior assessment: close-ended (on miswak use)	109 students; Islamic boarding school	85.3% were 15 years old	All miswak users (44.0% used miswak only, 56.0% used miswak in combination with other aid)	Awareness; OH practice
Gul et al., 2022 [51]	Cross-sectional	Pakistan	Self-administered questionnaires	75 medical and 45 dental students; university	19–21 years old	2.5% used miswak only, 20.8% used both miswak and toothbrush	Awareness; OH practice
Al-Shammari et al., 2007 [33]	Cross-sectional	Capital, Ahmadi, Hawalli, Jahra, Farwaniya, and Mubarak, Kuwait	Self-administered questionnaires	1925 adults; six governates	33.4 ± 9.0 years (18–70 years old)	33.0% used miswak occasionally or once daily	OH practice
Al-Tayar et al., 2019 [34]	Qualitative	Dawan Valley, Yemen	Structured interview	392 students; secondary schools	17.68 ± 1.27 years (15–21 years old)	43.1% used miswak	OH practice
Amin and Al-Abad 2008 [35]	Qualitative	Al-Hassa, Saudi Arabia	Structured interview	1115 male students; urban and rural primary schools	11.91 ± 1.0 years (10–14 years old)	44.6% used miswak	OH practice
Darout et al., 2005 [36]	Cross-sectional	Khartoum Province, Sudan	Self-administered questionnaires	396 students; secondary schools	12–22 years old	NA	OH practice
Farsi et al., 2004 [37]	Cross-sectional	Jeddah, Saudi Arabia	Self-administered questionnaires	2586 students; intermediate and high schools	12–18 years old	39.9% used miswak	OH practice
Azodo et al., 2010 [40]	Cross-sectional	Enugu, Nigeria	Self-administered questionnaires	242 dental students; university	75.2% were between 20–25-years old	51.7% used both miswak and toothbrush	OH practice
Clerehugh et al., 1995 [41]	Qualitative	Accra, Ghana	Structured interview (on OH practice)	177 students; secondary schools	14 years 4 months ± 2.77 months	8% used miswak only	OH practice
Varenne et al., 2006 [42]	Qualitative	Burkina Faso (West Africa)	Structured interview	505 children and 493 adults; urban and rural residence of Burkina Faso	Children: 12 years old Adults: age 35–44 years old	64.0% children and 76.0% adults used miswak	OH practice
Mlenga and Mumghamba, 2021 [43]	Cross-sectional	Lilongwe, Melawi	Self-administered questionnaires	409 students; urban dan rural primary schools	12.75 ± 1.15 years (11–14 years old)	24.9% used miswak	OH practice
Sajjad et al., 2018 [46]	Qualitative	Bhara Kahu, Pakistan	Structured interview	384 students and 36 teachers; primary schools	Children: 4–10 years old; teachers: NA	Children: 11.7% used miswak; Teachers: 47.22% used miswak as additional oral hygiene tool	OH practice
Waseem et al., 2015 [47]	Qualitative	Karachi, Pakistan	Structured interview	994 patients; dental outpatient department of a university	NA	43% used miswak	OH practice

OH: Oral hygiene; NA: Not applicable.

**Table 3:** Knowledge, awareness, and practice behavior related to *Salvadora persica* L. chewing stick (miswak) use.

Author and Year of Publication	Knowledge Related to *Salvadora persica* L.	Awareness Related to *Salvadora persica* L.	Practice Behavior Related to *Salvadora persica* L.
Che Musa et al., 2020 [48]	Low-to-moderate degree of knowledge in miswak use due to limited, restricted, and localized evidence of miswak as effective OH tool Respondents agreed that lack of information on how to use miswak effectively makes it less practiced at present	NA	NA
Nordin et al., 2014 [49]	91.6% knew miswak helps in oral healthcare >90% knew miswak was practiced by Prophet Muhammad PBUH, but only 19.7% knew the detailed information on its practices by Prophet Muhammad PBUH 52.9% knew biological properties of miswak help in the control of bacterial population—64.9% aware these had been confirmed by research and laboratory works 53.3% knew miswak must be soaked in water before use	Good perception towards miswak as a tool in oral healthcare: 58.7% agreed that using miswak in oral healthcare is suitable to practice in Malaysia 63.9% agreed that miswak is the best alternative to the toothbrush in oral healthcare for Malaysian Muslim population 71.1% agreed that miswak ensures the cleanliness of the mouth	NA
Almas et al., 2000 [28]	74.7% used miswak for prevention	NA	55.9% used miswak at prayer times, 48.8% used when felt mouth smell changes, 46.3% used after eating, 38.7% used with ablution, and 37.1% used upon waking up 77.4% used horizontal and vertical directions to clean tooth surfaces 74.9% cleaned all tooth surfaces with miswak
Darout et al., 2016 [3]	NA	Only 12.4% preferred the traditional way of cleaning teeth using miswak 50.7% used miswak as they felt better tooth cleaning with miswak	>50% of miswak users used miswak more than 2 times/day for more than 2 min
Al-Hammadi et al., 2018 [13]	NA	61.4% used miswak as primary OH method due to religious reasons, 7.3% used miswak due to scientific reasons After use of miswak, 84.7% feel fresh and teeth are whiter 76.9% respondents would advise their children to use both toothbrushing and miswak in their daily OH practice, 5.5% advised only miswak for their children	39.3% stored miswak for reuse in upper pocket exposed to air, 19.3% stored in upper pocket but covered, 23.9% stored in pocket after cutting off the used end 13.9% discarded miswak after single use 84.7% prefer to continue using miswak in combination with other teeth cleaning methods which may have more benefits
ALGhamdi et al., 2015 [23]	NA	Miswak was less likely to be used when students received OH education courses—students did not believe in miswak being the only tool for teeth cleaning	Males used both miswak and toothbrush at higher percentage (70.6%) than females (29.4%)
Almas et al., 2003 [29]	NA	Reasons for miswak use: Sunnah: 61.9% of males and 62.4% of females Better cleaning: 16.3% of males and 14.8% of females Freshness: 8.0% of males and 18.0% of females (*p* < 0.002)	59.0% of female teachers used miswak once/day, 47.0% male teachers used miswak more than 3 times/day (*p* < 0.001) No significant difference in methods of using miswak between gender (*p* = 0.189) No significant difference among different income groups on frequency of daily use of miswak (*p* = 0.382)
Al-Otaibi and Angmar-Mansson, 2004 [30]	NA	In military center: 64.5% used miswak for religious-only reasons, 7.5% used for OH reasons, 28.0% used due to both reasons In university center: 73.7% used miswak because of both religious dan hygiene reasons, 14.9% used as religious custom and 11.4% used miswak due to hygiene-only reasons	Regular miswak use was more frequent in males, older age groups, and in those with less education
Tubaishat et al., 2005 [31]	NA	51.0% of toothbrush users perceived toothbrush-plus-miswak the most effective in reducing plaque Level of education was associated with type of oral cleaning device used; toothbrushes and toothbrush-plus-miswak users were educated–holding baccalaureate or associate degrees	Toothbrush-plus-miswak users were most likely to spend 1–2 min each day cleaning their teeth and more likely to brush teeth twice/day
Vanka et al., 2021 [32]	NA	60.3% used miswak due to religious reasons, 15.5% due to scientific reasons, 8.6% due to cultural reasons, 3.4% due to cost, and 2.1% due to availability 54.3% felt their mouth was fresh after using miswak, 43.1% found their teeth to be whiter 88.8% would want the next generation to use a combination of miswak and toothbrush	6.0% chewed miswak for minutes, 18.1% chewed it until it turns bristle-likes 42.2% stored miswak by cutting the used end and storing it in a pocket
Agbor and Azodo 2013 [38]	NA	Miswak users believed the main reason for use is related to religion, and miswak has a positive effect in the mouth that cleans teeth better than the non-users	Miswak users were more frequent among males (88.1%) than females (72.1%) and increased with age
Hyder et al., 2023 [39]	NA	Reasons for using miswak: Religion (61.3%); *p* = 0.049 Better cleaning (38.7%)	51.6% used miswak 2 times/day, 32.2% used ≥3 times/day No significant association of education, monthly income and religion for the selection of tooth cleaning devices (*p* > 0.05)
Bramantoro et al., 2018 [50]	NA	Most of the respondents had positive attitudes toward miswak use: 87.0% respondents had positive attitude that miswak can help maintain oral and general health Lowest attitude (67.0%) was toward the ability of miswak to control oral bacteria growth	51.3% started using miswak when they enrolled in Islamic boarding school Miswak was mostly purchased at a store near the school (90.8%) 74.3% never shared miswak, 7.3% usually did share it between them
Fantaye et al., 2022 [44]	NA	Reasons for preferring miswak: Reasonable prices and proper cleaning: 71.0% totally and 50.0% partially visually impaired participants Religion: 4.4% totally and 5.0% partially visually impaired participants Practicality: 4.4% totally and 5.0% partially visually impaired participants	Regular use of miswak ≤2 times/day: 44.4% totally and 45% partially visually impaired participants Regular use of miswak ≥2 times/day: 26.6% totally and 15.0% partially visually impaired participants Use miswak for ≥3 min: 53.3% totally and 35% partially visually impaired participants 55.5% totally and 35% partially visually impaired applied the proper using miswak
Alayan et al., 2017 [45]	NA	8/8 participants reported religious reasons for their use of miswak 6/8 participants attributed their use of miswak to its bactericidal properties 8/8 participants perceived miswak is more effective at reducing plaque levels than a standard toothbrush 2/3 participants perceived miswak improved periodontal health and was less traumatic to gingiva than a standard toothbrush 7/8 participants reported miswak use had reduced notably since immigrating to New Zealand	All participants used the same method to prepare miswak–removing the twig’s bark at one end and then chewing the exposed stem to form bristles. The bristles were chewed until the desired softness was achieved Participants reported using miswak in a similar manner to a standard toothbrush, with miswak bristles used in circular motions and/or up-and-down against tooth surfaces 4/8 participants concentrated on brushing along the gingival margins of teeth 1/4 participants claimed greater time required to achieve plaque-free tooth surfaces than with conventional toothbrushing
Gul et al., 2022 [51]	NA	Perceived reasons of using miswak: religion (48.5%), culture (7.5%), scientific reasons (5%), practicality (4.2%) 70.0% preferred their families to use a combination of both toothbrush and miswak	36.0% stored miswak properly post-use
Al-Shammari et al., 2007 [33]	NA	NA	More males (43.1%) used miswak than females (22.6%); *p* < 0.001 33.0% used miswak occasionally or once/day
Al-Tayar et al., 2019 [34]	NA	NA	43.1% used miswak–significantly more males (55.4%) than females (31.0%), *p* < 0.001
Amin and Al-Abad 2008 [35]	NA	NA	Significantly higher (74.6%) miswak users were affected with caries than caries-free (*p* < 0.001)
Darout et al., 2005 [36]	NA	NA	64.3% males and 52% females used miswak ≥2 times/day 31.9% males and 35% females use rotational method, while the rest use other methods
Farsi et al., 2004 [37]	NA	NA	More males (80.8%) used miswak than females (2.9%) (*p* < 0.001) Miswak users were higher among governmental school students (72.3%) (*p* < 0.005) No significant difference related to age with respect to using miswak (*p* > 0.05)
Azodo et al., 2010 [40]	NA	NA	From 94.2% who practiced tongue cleaning, 2.1% used miswak for tongue cleaning
Clerehugh et al., 1995 [41]	NA	NA	No significant difference in teeth cleanliness based on gender or cleaning method (*p* > 0.05)
Varenne et al., 2006 [42]	NA	NA	More females were using miswak (*p* < 0.001) More miswak users in the rural areas compared to the urban areas (*p* < 0.001)
Mlenga and Mumghamba 2021 [43]	NA	NA	Significantly higher miswak users in rural (49.0%) than in urban (1.9%) schools (*p* < 0.001) Miswak users were significantly higher among pupils who had parents with primary (46.3%) education compared to those with secondary or higher (12.7%) education (*p* < 0.001)
Sajjad et al., 2018 [46]	NA	NA	52.3% of the children who used miswak admitted miswak was also used by family members
Waseem et al., 2015 [47]	NA	NA	Significant relation between socio-economic class and use of miswak (*p* < 0.01); majority of miswak users were in the unskilled occupation category

OH: Oral hygiene; NA: Not applicable.

## Data Availability

All studies used in this scoping review are included in this published article can be retrieved from https://figshare.com/articles/dataset/KAP_i_Salvadora_persica_i_Use/29877857 (accessed on 11 August 2025), which are also available from the corresponding author on reasonable request.

## Supplementary Materials

The following supporting information can be downloaded at: https://www.mdpi.com/article/10.3390/healthcare13212747/s1, Table S1: Findings of included studies on knowledge, awareness, and practices of miswak use in different populations.
